# Developing and evaluating a community‐driven intervention to promote uptake of HIV and contraception services among students enrolled in colleges and universities in Zimbabwe

**DOI:** 10.1002/jia2.26461

**Published:** 2025-06-26

**Authors:** Oppah Kuguyo, Lindiwe Mancitshana, Collin Mangenah, Mary K. Tumushime, Nancy Ruhode, Edward Matsikire, Jane Kalweo, Fern Terris‐Prestholt, Frances M. Cowan, Euphemia Lindelwe Sibanda

**Affiliations:** ^1^ Centre for Sexual Health and HIV/AIDS Research (CeSHHAR) Harare Zimbabwe; ^2^ Department of International Public Health Liverpool School of Tropical Medicine Liverpool UK; ^3^ Department of Global Health and Development London School of Hygiene and Tropical Medicine London UK; ^4^ UNAIDS Zimbabwe Harare Zimbabwe; ^5^ UNAIDS Geneva Switzerland

**Keywords:** community‐driven, HIV prevention, PEP, self‐care, sexual and reproductive health, young people

## Abstract

**Introduction:**

There is a growing appreciation that community‐led interventions are key to sustaining the HIV response and achieving HIV prevention and treatment targets. Together with young people in colleges/universities and Ministry of Health (MOH), we developed and evaluated a student‐led intervention for promoting the uptake of HIV self‐testing (HIVST), post‐exposure prophylaxis (PEP) and emergency contraception (EC) among college/university students.

**Methods:**

Over 3 months, in biweekly study team meetings, two workshops with students, two meetings with MOH, and a joint workshop with students, MOH and relevant stakeholders, we co‐developed an intervention for peer‐led promotion/distribution of HIVST, PEP, EC and condoms. The agreed intervention was piloted in three Zimbabwean colleges/universities from December 2023 to February 2024. Student peers distributed HIVST and condoms directly, and vouchers for PEP and EC that were redeemed at college/nearby clinics. During co‐development, students strongly preferred peer distribution of all commodities but this was restricted by regulatory requirements for PEP and EC. Peer distributors (*n* = 14) kept daily audio diaries of their experiences. In‐depth interviews were held with students (*n* = 18), peer distributors (*n* = 11) and key informants (*n* = 12) to explore views/preferences, with participant observations and four focus group discussions to provide additional insights. We determined the intervention development and implementation costs.

**Results:**

Peer‐led distribution of HIVST, PEP and EC to college/university students was acceptable, feasible, appropriate and generally implemented as intended. PEP and EC acceptability was driven by high HIV and pregnancy risk among students, who had no easy access to services. Of 100 PEP and 257 EC vouchers distributed, 30% and 40% were redeemed, respectively. The main barrier to PEP and EC uptake was moral judgement against premarital sex, which affected female students more. Judgemental health worker attitudes also limited uptake of PEP and EC. EC voucher redemption among female students was lower versus males, aOR = 0.4 (95% CI = 0.2−0.8), *p* = 0.019. Redemption was also higher at the college where the nearby clinic could be accessed discreetly. Total cost of the intervention per student was $14.57 (cross‐institution range: $7.26−$35.52).

**Conclusions:**

Student‐led distribution of HIVST, PEP and EC was feasible, acceptable and affordable. Making the intervention more community‐driven according to the 2024 WHO PEP guidelines will likely achieve great impact.

## 1 INTRODUCTION

Community‐led responses are actions/strategies informed by communities, and implemented by/for them to improve their health and human rights [1]. Communities are increasingly being recognized as key to driving sustained responses for achieving HIV prevention and treatment goals [2]. Correspondingly, in 2016, UN member states committed to ensuring that at least 30% of HIV service delivery is community‐led by 2030 [1]. Progress towards this is slow, with calls for research to inform how community‐led models, including youth‐led models, can be supported.

Youth‐led responses are important because young people lag behind in the uptake of HIV and sexual and reproductive health (SRH) services [3]. Across eastern, western, central and southern Africa (EWCSA), only 65% of people living with HIV aged 15−24 years know their HIV status, compared with 84% among adults >15 years [4]. Use of condoms, pre‐exposure prophylaxis (PrEP), post‐exposure prophylaxis (PEP) and voluntary medical male circumcision (VMMC) among young people is suboptimal [5, 6]. HIV incidence is high; about 160,000 women aged 15−24 years acquired HIV in EWCSA in 2022 [7], with incidence as high as 0.76% among adolescent girls and young women in Zimbabwe [8]. More than 80% of sexually active adolescents in EWCSA do not use contraception (14.6% unmet contraception need in Zimbabwe) [9], with millions facing unintended pregnancy, unsafe abortions and school drop‐out [10]. There is a high prevalence of spontaneous sex among young people, associated with the non‐use of condoms and contraception [11, 12]. This highlights the importance of emergency prevention for HIV and contraception, namely PEP and emergency contraception (EC), in addition to other combination prevention interventions. However, as stated above, these services are underutilized [13]. The recently updated World Health Organisation (WHO) PEP guidelines that endorse PEP delivery in communities, and through task sharing, will be important for driving uptake among young people [13].

Here, we describe a student‐led approach incorporating HIV self‐testing (HIVST) [14], and passive linkage (i.e. no active follow‐up to encourage linkage) to PEP and EC services for young people enrolled in colleges/universities. The approach promoted self‐care, where individuals take care of their health with/without health worker (HW) support [15, 16, 17, 18]. Self‐care is recommended by WHO as critical for achieving universal health coverage [15, 16, 17, 18]. We aimed to co‐develop and evaluate the acceptability, feasibility, adoption, appropriateness, fidelity and cost, of a student‐led self‐care intervention for promoting HIVST, PEP and EC uptake among college/university students.

## 2 METHODS

### 2.1 Study overview

**Figure 1:**
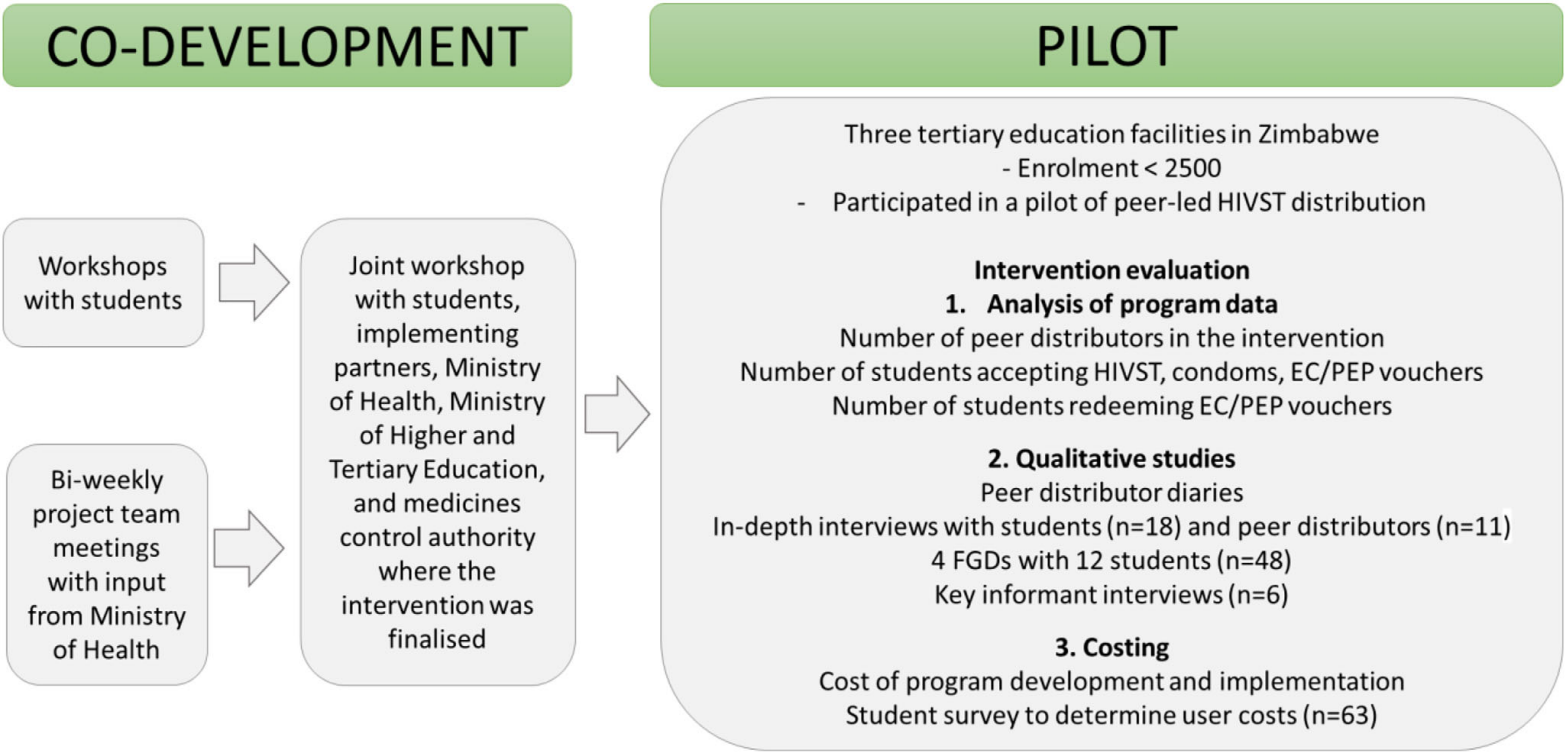
**Overview of the study**. Abbreviations: EC, emergency contraception; FGDs, focus group discussions; HIVST, HIV self‐test; PEP, post‐exposure prophylaxis.

Together with college/university students, Ministry of Health (MOH) and relevant stakeholders, we developed a student‐led self‐care intervention for HIV/SRH. Intervention development followed the WHO/Narasimhan self‐care framework that upholds human rights and gender equality while taking a people‐centred approach in an environment with health systems support and accountability [15, 18, 19]. We piloted the intervention in three colleges/universities over 1 month and used mixed methods to evaluate it (Figure 1).

### 2.2 Co‐development of the intervention

Our team comprised researchers and UNAIDS partners who worked on a completed trial (PACTR202111848628644) of peer‐led HIVST [14], where students requested an intervention that met their HIV prevention and contraception needs. Over 3 months, the team held biweekly meetings to develop a peer‐led intervention comprising HIVST, PEP and EC with insights from MOH (two meetings), and the medicines control authority (one meeting), Figure 1. We also held two workshops with students from the participating institutions to co‐develop the intervention. We used insights from the meetings and workshops to develop a preliminary intervention that was finalized in a joint co‐development workshop including students who participated in the above workshops, MOH and their partner implementers, and representatives of college/university leadership.

### 2.3 The agreed intervention

#### 2.3.1 Commodities to be distributed and distribution model

HIVST, PEP, EC, male and female condoms would be provided. Mobilization and commodity distribution would be peer‐led. Although students preferred collecting all commodities from peer distributors, national regulations restricted unlicensed personnel, such as students, from distributing PEP and EC. To address this, workshop participants agreed that peer distributors would distribute PEP and EC vouchers that could be redeemed through licensed HWs at college/nearby clinics. Voucher booklets were also securely placed at locations suggested by students (e.g. library, toilets) to give an option for self‐collection. HIVST, male and female condoms were distributed directly according to MOH guidelines. Students accessing HIVST received information on linkage to appropriate post‐test services.

#### 2.3.2 Documenting uptake of commodities

Basic demographic information would be collected during the distribution of condoms, HIVST, PEP and EC vouchers. Existing MOH registers would be used at clinics for documenting PEP and EC uptake.

### 2.4 Intervention pilot

We piloted the agreed intervention at three colleges/universities from December 2023 to February 2024. PEP, EC, male and female condoms and HIVST were provided by the study.

#### 2.4.1 Description of participating colleges/universities

The three colleges/universities participated in the preceding HIVST study [14]. They were purposively selected to include different institution types and geographic variation, with a maximum enrolment of 2500. The maximum enrolment (2500) was determined pragmatically. We included a Polytechnic college, University and Vocational college, with total enrolments of 1200, 2220 and 800 (Table 1). The Polytechnic college and University had onsite clinics where students redeemed EC and PEP vouchers, while students from the Vocational college redeemed the vouchers from a nearby public sector clinic.

**Table 1:** Characteristics of study colleges/universities

	Polytechnic college	University	Vocational college
Location	Small town about 100 km from the capital city (Harare)	Small town, about 90 km from Harare	Located in a high‐density residential suburb in Harare
Ownership	Government	Private (Christian)	Private (Christian)
Maximum enrolment	1200	2220	800
Subject area	Technical and vocational multi‐skills training. Offers national certificates, national diplomas, higher national diplomas and Bachelor of Technology	Degree‐level courses in law, commercial subjects, arts and education	Industrial training. Offers national diplomas, national certificates, national foundational certificates and short courses
Clinic location	Campus	Campus	Community
HIV services available at clinic	PrEP and ART initiation; Additional testing for positive self‐testing	PrEP and ART initiation; additional testing for positive self‐testing	PrEP, VMMC and ART initiation; Additional testing for positive self‐testing
Contraception services offered	Pill, injectable, emergency contraception, condoms^a^	Pill, injectable, emergency contraception, condoms^a^	Pill, injectable, implant, emergency contraception, condoms

Abbreviations: ART, antiretroviral therapy; PrEP, pre‐exposure prophylaxis; VMMC, voluntary male medical circumcision.

^a^ Although clinics technically offered condoms, they did not have condoms in stock during the study.

#### 2.4.2 Selection and training of peer distributors

At each college/university, students and college/university leadership identified potential peer distributors who were students aged ≥16 years and willing (written informed consent) to be distributors. Based on the previous peer‐led HIVST trial [14], we selected one peer distributor per 300 students, ensuring the representation of students who lived on/off campus. Before they started distribution, peer distributors were trained on (1) condom demonstration, (2) HIV testing and counselling according to MOH curricula, including how to demonstrate the correct use of HIVST and support people who are self‐testing, (3) educating people on PEP and EC, (4) ethical principles to uphold, (5) how to distribute and document uptake of commodities and/or vouchers, including eligibility criteria.

#### 2.4.3 Distribution of commodities during the pilot

At each college/university, distribution was done over 1 month. Eligibility criteria for receiving commodities and distribution considerations are summarized in Table 2. Students provided verbal consent to collect commodities, in line with local standard of care. Additional criteria specific to each commodity are summarized in Table 2. Distributors documented HIVST uptake in an mHealth app that collected information on age, sex and institution. For each EC and PEP voucher, the distributor kept a stub with recipient age, sex, level in college and institution (stubs were self‐completed for self‐collections). Distributors were trained to uphold privacy in all interactions. Distributors were paid a fixed stipend of US$50 after distribution ended.

**Table 2:** Commodity distribution procedures and criteria

Commodity	Quantity distributed at a time	Inclusion criteria	Explanation from peer distributors
HIVST	1	Student enrolled at participating college/university Not aware of HIV status	*Strong emphasis that those who knew their HIV status and were on HIV treatment should not use self‐test, with warning of risk of false negatives explained*.
PEP vouchers	1	Student enrolled at participating college/university Self‐reported HIV‐negative status Had recent (<72 hours) unprotected sex with a person living with HIV or someone of unknown HIV status and suspected to be at high risk of HIV	*PEP needed to be taken as soon as possible after unprotected sex, and no later than 72 hours*.
EC vouchers	1	Student enrolled at participating college/university Female student^a^ Not on a current family planning method Had recent (<72 hours) unprotected sex	*EC needed to be taken as soon as possible after unprotected sex, and no later than 72 hours*
Condoms^b^	5	Student enrolled at the facility Students enrolled at the institution	

Abbreviations: EC, emergency contraception; HIVST, HIV self‐testing; PEP, post‐exposure prophylaxis.

^a^ During implementation, distributors had requests by boyfriends to collect vouchers on behalf of their girlfriends, and this was allowed.

^b^ Both male and female condoms.

### 2.5 Evaluation of the intervention

Using Proctor's Framework [20], we analysed programme data, conducted cost surveys and used qualitative studies to evaluate the intervention for: acceptability, adoption (uptake), appropriateness, feasibility, fidelity and costs. Table 3 highlights the methods used for each outcome.

**Table 3:** Proctors’ outcomes and evaluation methods

Outcome	Indicators	Evaluation methods
Acceptability	% invited distributors accepted participation	Analysis of programme data
	% invited distributors completed training	Analysis of programme data
	Acceptability themes from qualitative research	Peer distributor audio diaries Peer distributor in‐depth interviews Participant observation Student in‐depth interviews Student focus group discussions Key informant interviews
Adoption (uptake)	% Students accepting HIVST % Students accepting PEP % Students accepting EC % Students accepting male/female condoms % Students redeeming PEP vouchers % Students redeeming EC vouchers	Analysis of programme data
	Themes from qualitative studies related to adoption	Peer distributor audio diaries Peer distributor in‐depth interviews Participant observation Student in‐depth interviews Student FGDs Key informant interviews
Appropriateness, feasibility, fidelity	Themes from qualitative studies	Peer distributor audio diaries Peer distributor in‐depth interviews Participant observation Student in‐depth interviews Student focus group discussions Key informant interviews
Costs	Cost of intervention development	Programme data
	Cost of intervention provision Cost per student enrolled	Programme data

Abbreviations: EC, emergency contraception; FGDs, focus group discussions; HIVST, HIV self‐testing; PEP, post‐exposure prophylaxis.

### 2.6 Programme data

We descriptively computed Acceptability and Adoption outcomes in Table 3. Univariable and multivariable logistic regression was used to determine factors associated with redemption of PEP and EC. For the outcome variables (% redemption of PEP; % redemption of EC), the numerators were numbers of students who redeemed PEP or EC and the denominators were numbers of students who collected PEP and EC vouchers, respectively. For adjusted analyses, age, sex, level in college and institution were built into the same logistic regression model. We verified that there was no collinearity between age and level in college. Programme data were analysed using STATA v17.0 [21].

### 2.7 Costing

We estimated full annual economic costs including actual (financial) expenses and non‐financial costs (student distributors’ time and materials, other donated inputs and cross subsidization by pre‐existing health programmes) for resource inputs consumed during distribution. Costs were classified according to the rollout stage—(1) pre‐implementation (meetings and workshops), (2) start‐up, for example training, (3) implementation and (4) type (capital and recurrent).

#### 2.7.1 Costing analysis

To ensure that only the value of capital items used during the project lifetime were included, pre‐implementation, start‐up and other capital costs were annualized based on appropriate useful lifespans and at a 3% discount rate. Interviews with programme implementation team members helped disaggregate and allocate staff time to relevant activities. Distributors’ time was valued based on the fixed stipend of US$50 paid after distribution. Input costs, on‐site observations, and monitoring and evaluation data were used to estimate total programme economic costs of product distribution. Cost per student was estimated by dividing programme cost by student enrolment per institution. Costs were estimated in 2023 US dollars. Data management and analysis was conducted in Microsoft Excel^®^.

### 2.8 Qualitative studies

As indicated in Table 3, we used various qualitative methods to explore acceptability, adoption, appropriateness, feasibility and fidelity (whether intervention was implemented as intended). For distributors, we recruited all who were willing/able to participate in qualitative studies; for other participant types, we conducted recruitment until theoretical saturation was reached [22].

- *Participant observations*: Trained social science researchers observed student behaviour and interactions about the intervention. Observations were made during distributor training and at two support visits to each college/institution according to a guide soliciting impressions on distributor comprehension of training, distributor enthusiasm, acceptability of the intervention among students and fidelity to implementation.
- *Audio diaries among distributors (n = 14)*: Trained distributors were asked to make audio records depicting their experiences from the start of distribution to 2 weeks after the end of distribution. Social science researchers provided training on how to make audio records on tablets, including examples of experiences and impressions to record, and ethical principles to uphold (e.g. upholding confidentiality by not mentioning people's names in audio recordings).
- *In‐depth interviews (IDIs) among distributors (n = 11)*: One month after the distribution was completed, distributors were interviewed in‐depth to explore experiences with distribution.
- *IDIs among students (n = 18)*: One month after distribution ended, students were interviewed in‐depth to explore user experiences, acceptability of the intervention, and barriers and facilitators to uptake of the different commodities. Purposive selection was done to ensure balance by sex, study year group and uptake/non‐uptake of commodities.
- *Focus group discussions (FGDs) among students (4 FGDs, n = 45)*: One month after implementation, four FGDs with 11−12 students per group were held with students who accepted/did not accept commodities to explore insights at group level.
- *Key informant interviews (n = 12)* MOH representatives at central and local levels, and MOH implementing partners in the fields of HIV and contraception were interviewed in‐depth about views on the intervention and how it can be improved.

IDIs, FGDs and key informant interviews were facilitated by trained social science researchers using discussion guides and were audio‐recorded. Analysis was thematic [23]; commencing together with data collection with field notes for each audio diary, interview/discussion focused on emerging themes. The research team had regular discussions to interrogate qualitative findings, their relationship with programme data and to inform further exploration. After data collection was complete, analytic summaries of each theme drew comparisons within and across participants and data collection methods. These summaries were used to develop coding frameworks that were used for coding the data in NVIVO 11 [24].

### 2.9 Community‐driven nature of the study

**Figure 2:**
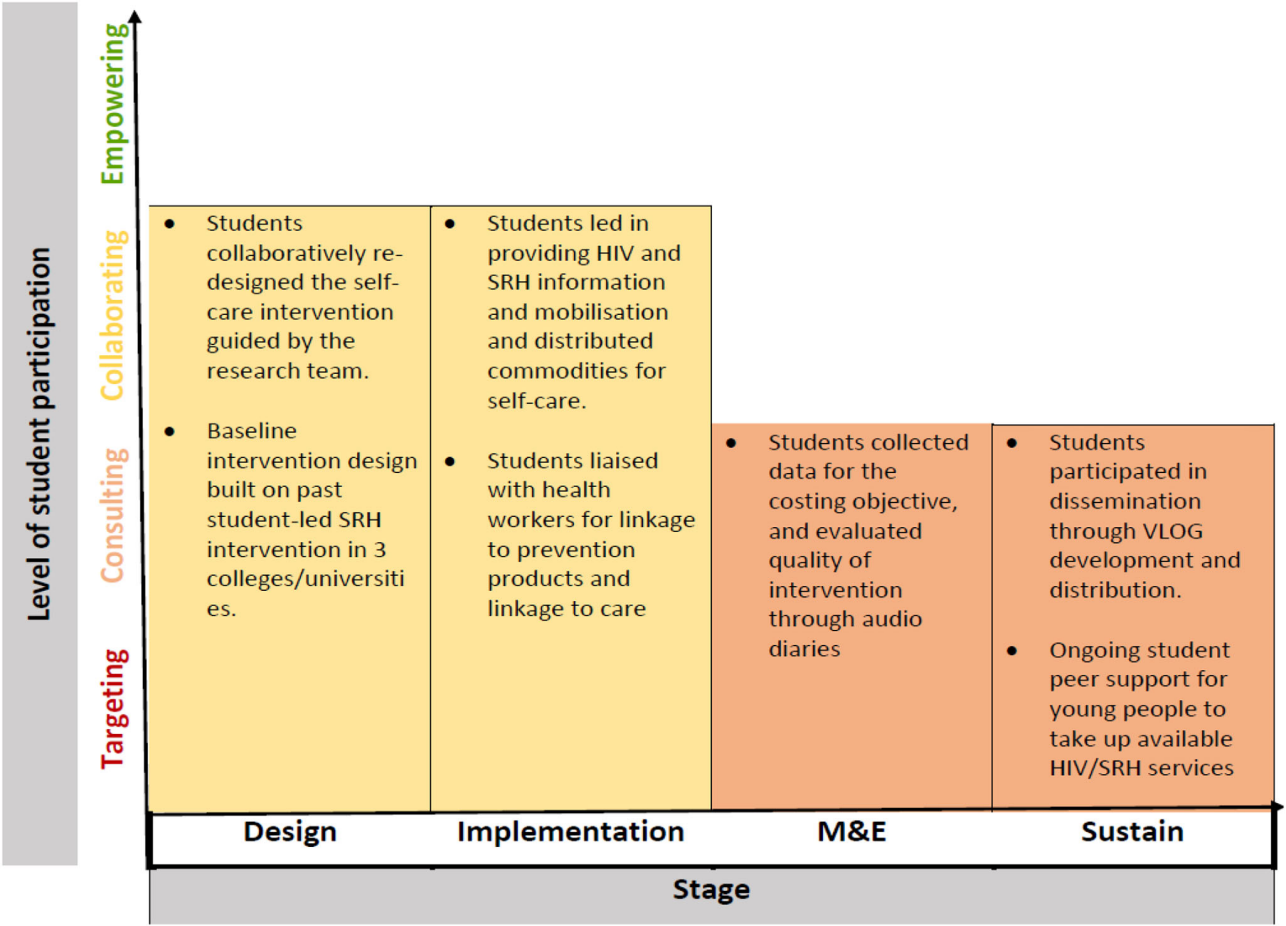
**An illustration of the level of student participation in the different stages of the self‐care intervention in accordance with the McGee et al. [25] framework for qualifying community‐led interventions**. Abbreviations: M&E, monitoring and evaluation; SRH, sexual and reproductive health; VLOG, video blog.

Various components of the study were student‐driven, including intervention conception, development and implementation. Figure 2 summarizes the extent to which the different components were student‐driven.

### 2.10 Ethical considerations

Ethical approval was obtained from the national ethics committee, Medical Research Council of Zimbabwe (Ref MRCZ/A/2971). Written informed consent was obtained before participation in all qualitative studies.

## 3 RESULTS

### 3.1 Programme data analysis

#### 3.1.1 Acceptability

**Figure 3:**
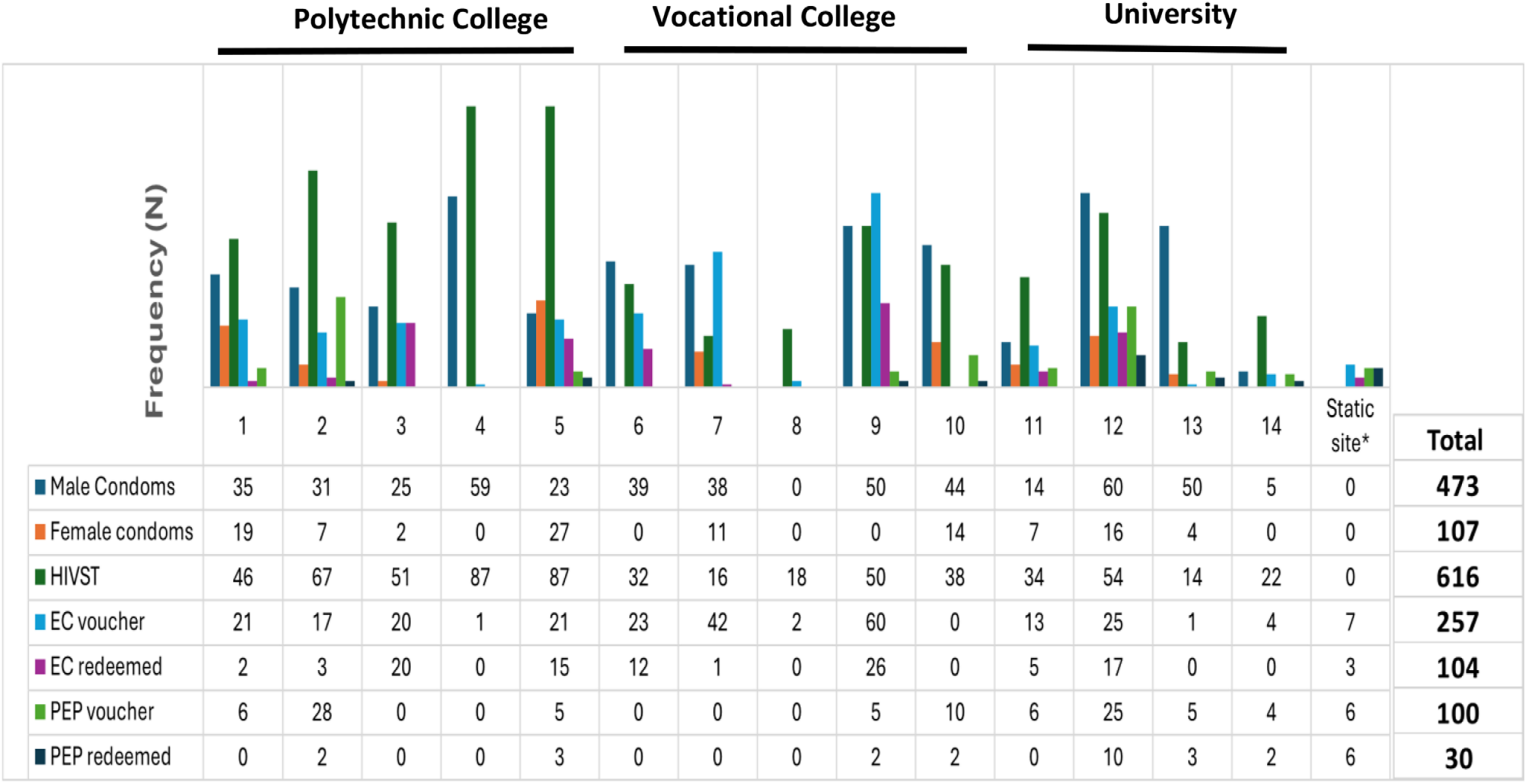
**Commodities distributed stratified by peer distributor (1−14) and institution**. Abbreviations: EC, emergency contraception; HIVST, HIV self‐testing; PEP, post‐exposure prophylaxis. *Indicates vouchers for PEP and EC that were self‐collected from public points such as the library, auditorium and toilets rather than collected from a distributor.

Across the three institutions, 17 students who were suggested by their colleges were invited to participate as distributors, and all accepted participation. Of these, 15 (88.2%) completed training, and 14 (93.3%) started and completed distribution, Figure 3.

#### 3.1.2 Adoption

Of 4220 enrolled students across the three colleges, 2896 students were present during the pilot (the rest were away on scheduled industrial attachments). Across the three institutions, 473 and 107 students accepted male and female condoms, respectively, 616 accepted HIVST, and 257 and 100 accepted EC and PEP vouchers, respectively, Figure 3.

The median (IQR) age of students accepting male condoms was 22 (20−24) years, female condoms: 21 (20−23) years, HIVST: 22 (17−24) years, PEP vouchers: 23 (21−25) years and EC vouchers: 21 (19−23) years (Table 4). Both males (*n* = 118; 45.9%) and females (*n* = 108; 42%) collected EC vouchers, and a greater proportion of first‐year students (*n* = 109; 42.4%) collected EC vouchers compared to other college levels (Table 4). This was mostly driven by the first‐year students at the vocational college (Table S1).

**Table 4:** Characteristics of students taking up commodities

Characteristic	Male condom (*N* = 473)	Female condom (*N* = 107)	HIVST (*N* = 616)	PEP voucher (*N* = 100)	EC voucher (*N* = 257)
**Age (median [IQR])**	22 (20−24)	21 (20−23)	22 (17−24)	23 (21−25)	21 (19−23)

	*n* (*n*/*N*%)	*n* (*n*/*N*%)	*n* (*n*/*N*%)	*n* (*n*/*N*%)	*n* (*n*/*N*%)
**Age range in years**					
16–19	62 (13.1)	16 (15.0)	91 (14.8)	3 (3.0)	57 (22.2)
20–24	327 (69.1)	77 (72.0)	382 (62.0)	63 (63.0)	135 (52.5)
25–29	77 (16.3)	14 (13.0)	130 (21.1)	26 (26.0)	33 (12.8)
30 +	7 (1.5)	0 (0.0)	13 (2.1)	0 (0.0)	1 (0.4)
Missing	0 (0.0)	0 (0.0)	0 (0.0)	8 (8.0)	31 (12.1)
**Sex**					
Male	418 (88.4)	25 (23.4)	307 (49.8)	51 (51.0)	118 (45.9)
Female	47 (9.9)	82 (76.6)	309 (50.2)	41 (41.0)	108 (42.0)
Missing	8 (1.7)	0 (0.0)	0 (0.0)	8 (8.0)	31 (12.1)
**Level in college**					
First	154 (32.6)	34 (31.8)	−^a^	18 (18.0)	109 (42.4)
Second	153 (32.3)	44 (41.1)	−^a^	34 (34.0)	64 (24.9)
Third	126 (26.6)	22 (20.6)	−^a^	23 (23.0)	33 (12.8)
Fourth and over	30 (6.3)	7 (6.5)	−^a^	12 (12.0)	17 (6.6)
Missing	10 (2.1)	0 (0.0)	−^a^	13 (13.0)	34 (13.2)
**Institution**					
Polytechnic college	173 (36.6)	55 (51.4)	338 (54.9)	45 (45.0)	81 (31.5)
Vocational college	127 (26.8)	11 (10.3)	116 (18.8)	5 (5.0)	128 (49.8)
University	173 (36.6)	41 (38.3)	162 (26.3)	50 (50.0)	48 (18.7)

Abbreviations: EC, emergency contraception; HIVST, HIV self‐testing; IQR, inter‐quartile range; PEP, post‐exposure prophylaxis.

^a^ Indicates that the tool used for documenting HIVST distribution did not collect this data.

**Figure 4:**
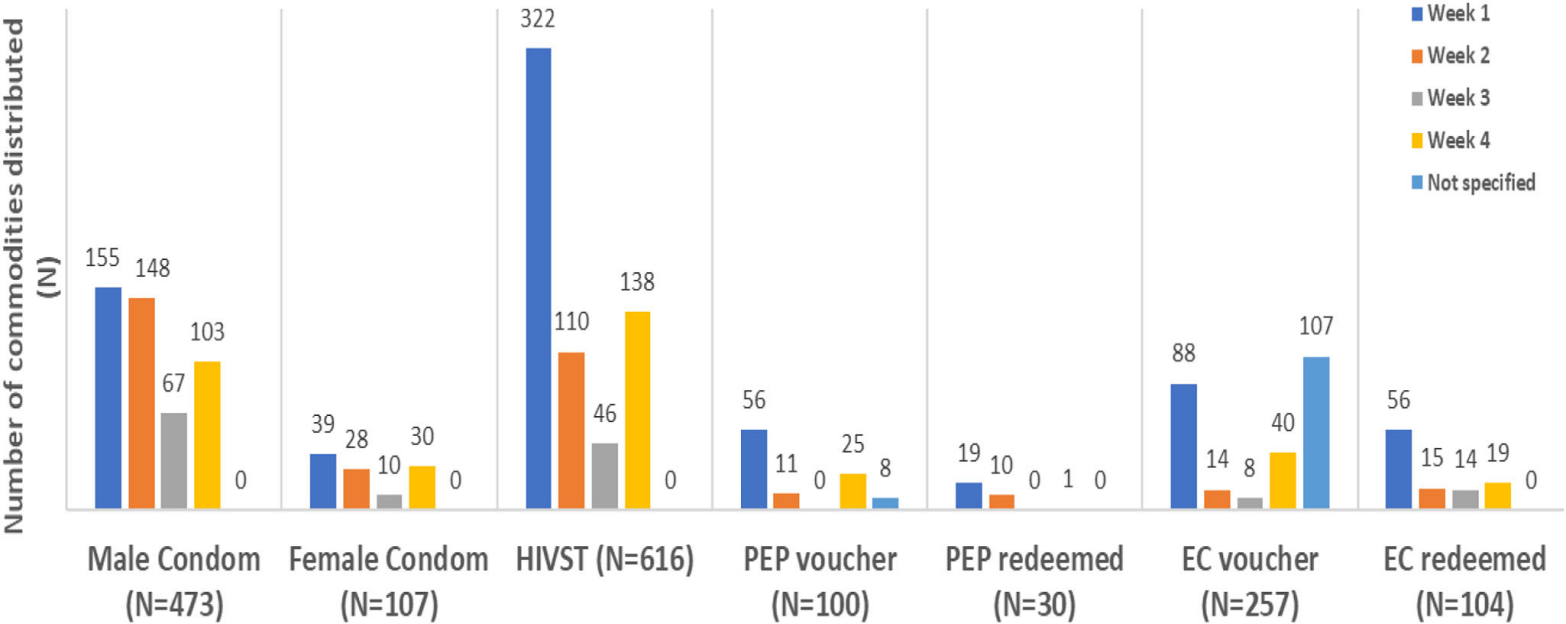
**Number of commodities distributed in the intervention pilot, per week**. Abbreviations: EC, emergency contraception; HIVST, HIV self‐testing; PEP, post‐exposure prophylaxis.

The highest uptake of all commodities happened in week 1 of implementation, with a general dip in weeks 2 and 3 and an increase in week 4, Figure 4.

##### 3.1.2.1 Redemption of PEP vouchers

Of the 100 PEP vouchers distributed, 30 (30.0%) were redeemed. Redemption did not differ by age, sex or year group (Table 5). There was a tendency towards a difference of PEP redemption by institution: at the polytechnic college, 11 (24.4%) students who collected PEP vouchers redeemed them, compared with 34% at the vocational college, adjusted odds ratio (aOR) 3.9 (0.9–17.4), *p* = 0.075 and 40% at the University, aOR 3.6 (0.3–28.9), *p* = 0.287. Of note, all six PEP vouchers that were self‐collected (rather than collected from a peer) were redeemed.

**Table 5:** Factors associated with redeeming PEP or EC among students who accepted vouchers

	Characteristic	Frequency *n*(*n*/*N*%)	Redemptions *n*_1_ (*n*_1_/*n*%)	Unadjusted OR (95% CI)	*p*	Adjusted OR (95% CI)	*p*
PEP (*N* = 92)	Age			0.9 (0.7–1.1)	0.356	0.9 (0.6–1.3)	0.540
	**Age range in years**						
	16−24	66 (71.8)	18 (28.6)	1.7 (0.5–5.1)	0.363		
	25−29	26 (28.3)	5 (19.2)	Ref.	Ref.		
	**Sex**						
	Female	41 (44.6)	9 (22.0)	Ref.	Ref.	Ref.	Ref.
	Male	51 (55.4)	14 (27.5)	1.3 (0.5–3.5)	0.546	1.6 (0.5–5.1)	0.396
	**Level in college**						
	First	18 (19.6)	5 (26.3)	Ref.	Ref.	Ref.	Ref.
	Second	34 (37.0)	7 (21.2)	0.8 (0.2–2.8)	0.675	0.5 (0.1–2.4)	0.387
	Third	23 (26.4)	6 (26.0)	1.0 (0.3–4.0)	0.987	1.5 (0.2–11.6)	0.713
	Fourth and above	12 (13.8)	5 (41.7)	2.0 (0.4–9.3)	0.376	1.3 (0.1–12.6)	0.541
	**Institution**						
	Polytechnic college	45 (48.9)	11 (24.4)	Ref.	Ref.	Ref.	Ref.
	Vocational college	50 (54.3)	17 (34.0)	1.6 (0.6–3.9)	0.309	3.9 (0.9–17.4)	0.075
	University	5 (5.4)	2 (40.0)	2.1 (0.3–13.9)	0.459	3.6 (0.3–28.9)	0.287
EC (*N* = 226)	Age			1.0 (0.9–1.20)	0.490	1.0 (0.9–1.1)	0.811
	**Age range in years**						
	16−19	57 (25.2)	14 (24.6)	Ref.	Ref.		
	20−24	135 (59.7)	51 (37.8)	1.9 (0.9–3.7)	0.085		
	25+	34 (15.0)	8 (23.5)	0.9 (0.3–2.6)	0.911		
	**Sex**						
	Male	118 (52.2)	46 (40.0)	Ref.	Ref.	Ref.	Ref.
	Female	108 (47.8)	27 (25.0)	0.5 (0.3–0.9)	0.026	0.4 (0.2–0.8)	0.019
	**Level in college**						
	First	109 (48.9)	32 (29.4)	Ref.	Ref.	Ref.	Ref.
	Second	64 (28.7)	18 (28.1)	1.1 (0.5–2.1)	0.863	1.3 (0.6–2.8)	0.522
	Third	33 (14.8)	14 (42.4)	1.8 (0.8–4.0)	0.163	2.1 (0.8–5.7)	0.128
	Fourth and above	17 (17.6)	9 (52.9)	2.7 (1.0–7.6)	0.060	1.3 (0.3–5.0)	0.715
	**Institution**						
	Polytechnic college	81 (31.5)	41 (50.6)	Ref.	Ref.	Ref.	Ref.
	Vocational college	48 (18.7)	24 (50.0)	1.0 (0.5–2.0)	0.946	3.5 (1.2–10.2)	0.021
	University	128 (49.8)	39 (30.5)	0.4 (0.2–0.8)	0.004	0.9 (0.3–2.7)	0.828

##### 3.1.2.2 Redemption of EC vouchers

Out of the 257 EC vouchers distributed, 104 (40.4%) were redeemed. Twenty‐seven (25%) female students redeemed EC vouchers compared to 46 (40%) male students (aOR = 0.4 95% CI = 0.2−0.8; *p* = 0.019) (Table 5). Redemption of EC was also higher at the vocational college compared to the Polytechnic College, aOR 3.5 (1.2–10.2), *p* = 0.021.

### 3.2 Costing results

Table 6 summarizes the programme and unit costs by college/university. The total programme costs were $42,205 (cross‐institution range: $13,674−$14,598) when the value of distributor time is based on the $50 incentive paid after distribution. Dividing total programme cost by number of students exposed to the intervention *(Students on campus during 4‐week intervention period)* yielded cost per student of $14.57 (cross‐institution range: $7.26−$35.52). However, and for the purposes of budgeting/planning for wider student reach, dividing total programme cost by total student enrolment *(including students who were away on industrial attachment during the intervention)* yielded cost per student of $10.00 (cross‐institution range: $6.28−$17.09). Most (98%) costs were recurrent implementation inputs, with programme personnel (54%) and supervision (31%) as the main cost drivers.

**Table 6:** Programme and unit cost summary by implementation stage

Cost categories $ (by input)	Polytechnic college	Vocational college	University	Total
**Intervention development**	***Cost ($)***	***Cost ($)***	***Cost ($)***	***Cost ($)***
*Sensitization (students)*	$5	$5	$5	$15
*Consultation meeting with stakeholders*	$175	$175	$175	$175
**Total capital cost—Intervention development**	$190	$190	$190	$190
**Implementation**				
*Initial training*	$281	$227	$58	$566
**Total capital cost—Implementation**	$281	$227	$58	$566
**Capital costs**				
**Intervention—Implementation**				
*Sensitization (students)*	$5	$5	$5	$15
*Joint workshop*	$58	$58	$58	$175
*Initial training*	$281	$227	$58	$566
**Total capital cost—Implementation**	$344	$290	$122	$756
**Recurrent costs—Implementation**				
*Personnel HQ*	$4295	$4440	$4368	$13,103
*Personnel (programme)*	$7539	$7540	$7540	$22,619
*Personnel (student distributors)*	$250	$250	$250	$750
*Supplies (distributors)*	$71	$71	$71	$214
*Supplies (products)*	$1359	$525	$700	$2583
*Visits (support and supervision + closeout)*	$468	$319	$479	$1266
*Communications*	$45	$45	$45	$135
*Vehicle operation and maintenance*	$31	$31	$31	$92
*Building operation and maintenance*	$100	$103	$101	$304
*Other recurrent*	$96	$99	$97	$292
Total recurrent cost—Implementation	$14,254	$13,384	$13,811	$41,449
Total programme cost	$14,598	$13,674	$13,933	$42,205
Total # students exposed^a^	591	385	1920	2896
*Cost per student enrolled*	$24.70	$35.52	$7.26	$14.57
Total student enrolment^b^	1200	800	2220	4220
*Cost per student enrolled* (total enrolment)	$12.17	$17.09	$6.28	$10.00

Abbreviation: HQ, head quarter.

^a^ # students exposed—students on campus during 4‐week intervention period.

^b^ Total student enrolment—for budgeting/planning for wider reach.

### 3.3 Qualitative study results

#### 3.3.1 Acceptability and adoption (uptake)

There was high acceptability and a great need for HIV and pregnancy prevention services among students. Across all data collection methods, students, distributors, lecturers and HWs said PEP and EC provision for students was long overdue. It was widely discussed that students needed these services but had no easy access. Although both college/university clinics reportedly offered condoms, none were available during the pilot.

“…here they don't allow distribution of condoms because they say it's a Christian institution, so the children have no access” (KII, 38‐year‐old female, HW).

Distributors reported that even before their distributor training was complete, they were overwhelmed by requests for products and information—this was confirmed during participant observations where some students requested immediate EC and PEP voucher access because the windows since unprotected sex were closing. Distributors reported rapid uptake of PEP, EC, HIVST kits and condoms soon after distribution began (Figure 4).

The main driver of acceptability of HIV and pregnancy prevention services was the high risk for HIV, sexually transmitted infections (STI) and pregnancy among students, which was acknowledged across all data collection methods. Many students reportedly engaged in condomless sex and had concurrent sexual partners within and outside college, including transactional sex partners. Distributors noted that before the intervention, in their role as peer educators/counsellors, they frequently interacted with students suffering from STIs with no access to treatment, with many reports of unintended pregnancies and abortions. Participant observers and students described “semester marriages” where students moved in together to save accommodation/living costs, with reports that condom use in such partnerships was rare. In such “stable” relationships, condom use was thought a sign of partner distrust: *“wearing a condom against a partner is accusing them of witchcraft.”* Students reportedly often found themselves in situations where they had spontaneous sex, where condom use was less likely—students reported that this made PEP invaluable.

“When this program came and they introduced PEP to us, these things are very important especially to students because there is a lot of mischief” (IDI, 27‐year‐old male, Peer distributor 4)

##### 3.3.1.1 Views on peer‐driven distribution

Acceptability and adoption were also motivated by the peer‐driven nature of the intervention, which students reported leveraged the influence, trust and non‐judgemental nature of existing peer relationships. Having the peer distributors in proximity also facilitated easy uptake.

“I think if (PEP) is distributed by these young distributors, it will be better because we are able to talk to them and say, ‘mate things are not well, this is what happened, so I want PEPP” (IDI, 24‐year‐old female, Student).

Key informants were also supportive of peer‐led distribution. However, some worried that the quality of service provided by peers may be suboptimal, with assertions that peer distributors lacked specialized training and had the potential to misuse products in their custody. They worried that peer distributors may have problems upholding confidentiality, although no reports of breached confidentiality were made. A few students also raised concerns about confidentiality and suggested including distribution by lecturers, which they said would expand the choices available for students who worried about lack of confidentiality among peers.

“I think the morning after program needs to be administered by an older person who is not a student, maybe a lecturer. Because students have a challenge that even the one who is distributing, you don't know to what extent they can maintain confidentiality…” (FGD, 18‐year‐old, Female student).

##### 3.3.1.2 Barriers to uptake of commodities

Moral judgement related to premarital sex was a major barrier to the uptake of services. This is a deeply enshrined societal judgement across Zimbabwe. Although reports showed it was perceived everywhere, students in Christian‐owned institutions worried about potential judgement from lecturers, nurses and other college staff who were seen as proponents of Christian values that discourage premarital sex. Although students felt comfortable collecting PEP and EC vouchers from peers, fear of moral judgement made it challenging for them to openly redeem vouchers from clinics. At two colleges, the walkway to the clinic and clinic entrance were clearly visible from lecturers’ and/or Christian leaders’ residences, which amplified the difficulty. Female students reportedly worried about moral judgement more than male students; some female students felt too shy to collect any of the commodities, even from peer distributors. Male partners, keen to prevent unintended pregnancy, were reported to play active roles in collecting EC vouchers and redeeming them for their shy girlfriends. This was confirmed by the programme data which showed that 40% of male students who collected EC vouchers redeemed them compared to 25% among females.

“You could be seen going there to take it (PEP) so it was not safe for students to go there just because you would go to the clinic in daylight from 8 to 4… because our clinic is in the open.” (IDI, 27‐year‐old male‐Peer distributor 2).

“…Girls were particularly shy to go and collect morning after, so boys did it for them…” (20‐year‐old female, Peer distributor 14)

Age differences between HWs and students created a significant barrier for students to redeem PEP/EC. Students perceived HWs to be “*adult‐like,”* which made it difficult to admit to being sexually active, let alone without condoms.

“So for someone to make the initial decision to go and see the elderly nurses who are as old as one's father or mother, or for someone to clearly tell their story, haa I don't think one will feel comfortable” (IDI, 24‐year‐old, Female student).

Students notably reported that HWs exhibited judgemental attitudes, lack of empathy, and were mean and unwelcoming towards students redeeming PEP/EC vouchers. They reportedly used demeaning words and shouted at students for engaging in premarital sex or unprotected sex. In some cases, HWs threatened to or refused to give commodities to discourage students from continued “risky” behaviour.

“The nurse said, but you can't just come and tell me that, so you are going to have to get it (HIV)…I can just deny you (the PEP), then you will get it, the disease.” (FGD, 24‐year‐old, Female student).

“…haa the nurses can embarrass you, especially if you try to use a low voice (to prevent other people hearing), they will immediately shout, saying ‘uhh, you recklessly decided to have wet sex (unprotected sex), that's why you want morning after’.” (FGD, 22‐year‐old, Female student).

Students underscored the need for peer distributors to provide both PEP and EC directly, rather than vouchers, to evade the negative interactions with HWs. Some lecturers also supported this notion.

Pill burden associated with PEP was also a barrier, which interacted with moral judgement. Students shared that because PEP involves a lengthy medication course, it can be overwhelming and increase the likelihood of others noticing. Activities that happen during the 28‐day period could discourage continuation, for example some students reported that if they had to go home during the 28‐day period they would stop taking PEP so that parents would not find out.

“…the (28‐day) course may be a challenge…if people see you taking it (PEP)…one may miss doses over the 28 days. Maybe they go home over the weekend so they decide to pause, or another time they may fear that their roommate has seen them and decide to stop.” (IDI, 25‐year‐old, Female student)

However, lived experiences from students who took‐up PEP demonstrated commitment to prevail over adherence challenges.

“During the first days it was not easy because I was not used to taking medication. So, as you keep taking it, you end up getting used to it” (IDI, 23‐year‐old, Female student)

Another barrier was reluctance to undergo provider‐delivered HIV testing, a prerequisite for accessing PEP according to national guidelines. Students who received PEP vouchers had generally also received HIVST and tested themselves; they reported feeling that the additional testing was a waste of time. Some students feared an HIV‐positive result.

“So, think about the whole process where I start by getting tested using the blood thing, then what, yet I already tested myself. For me to get tested, then wait, that's why I didn't return to go and collect what's‐the‐name, the PEP…” (IDI, 22‐year‐old, Female student)

#### 3.3.2 Feasibility, appropriateness and fidelity to implementation

Participant observations and programme data showed that peer distributors implemented the intervention as expected. Distributors confirmed this, reporting that they enjoyed the work and found it feasible to do. HWs reported facilitating PEP and EC redemption as expected. However, as described above, students reported judgemental and unfriendly treatment when seeking PEP and EC vouchers. Most HW narratives painted their work in a good light, highlighting *“provision of counselling to discourage risky behaviour.”*

The intervention provided a 28‐day course of PEP following potential exposure to HIV. Provision of PEP for future exposures (PEP‐in‐pocket) was not part of the intervention. However, we found students collecting PEP/EC for future use, highlighting acknowledgement of HIV and pregnancy risk and acceptability of the intervention. Desire for PEP‐in‐pocket was associated with knowledge that the programme was coming to an end, and students wanted to be sure they got emergency stocks before the opportunity lapsed. There was an indication that in an emergency, PEP‐in‐pocket was useful for starting early within the 72‐hour window as it avoided lengthy and dreaded processes before collection (HIV testing).

“I then had strong desire to ensure that I always have PEP because tomorrow is unknown, to be honest(chuckles)” (24‐year‐old, Female student).

“What if we keep the PEP, so that if there is an emergency, I just take it. The problem is that nurses want us to get tested, but if the young person already has their PEP in the room, they will just take it.” (IDI, 20‐year‐old female, Peer distributor 5).

There were indications of fears of risk compensation during intervention implementation. HWs, lecturers across all institutions, some key informants and students reported that PEP and EC availability appeared to encourage unprotected sex among students. Narratives by lecturers, HWs and other key informants were hypothetical on this, although some students and peer distributors said they knew this was happening. One student confessed that his girlfriend encouraged him not to wear a condom as she could use the morning after pill afterwards.

“…it made students indulge in unprotected sex instead of abstinence… So, students were no longer using condoms thinking ‘we will take PEP anyway” (IDI, 27‐year‐old male, Peer distributor 2).

## 4 DISCUSSION

In this mixed‐methods study, we found that a co‐developed self‐care intervention with peer‐led distribution of HIVST, PEP and EC to college/university students was acceptable, feasible, appropriate, and generally implemented as intended. Acceptability of PEP and EC was driven by unmet need—there were reports of high HIV and pregnancy risk among students, yet there was no easy access to HIV prevention and contraception services. Although students preferred to have PEP and EC distributed directly by peers, regulatory requirements did not support this, hence student peers distributed vouchers that were redeemed at nearby clinics. Of the PEP and EC vouchers that were distributed, 30% and 40% were redeemed. The main barrier to PEP and EC uptake was moral judgement associated with premarital sex, which affected female students more than males. Unfriendly HW attitudes limited the uptake of PEP and EC.

The high HIV risk we reported is in line with global data for young people [26, 27, 28, 29, 30]. It is worrying that despite this high risk, there is poor access to HIV and SRH services, which has also been reported in other settings [31]. Poor condom access in study communities in 2024 is shocking. Condom use and education has decreased among young people globally amid cuts in condom promotion budgets [32]. There is an urgent need to revitalize this, ensuring that this includes condom negotiation skills alongside implementation of combination prevention programmes that include PEP to PrEP or PrEP to PEP transitions and optimum access to contraception services.

Provision of services needs to address barriers uncovered in this study, including moral judgement for premarital sex and unfriendly/judgemental attitudes of HWs. At the vocational college, redemption of vouchers was likely higher because the clinic is separate from the college, so students could access the clinic without fear of being seen by their peers and lecturers. Investing in youth friendly programmes that uphold confidentiality and discreetness may be key to optimizing uptake of HIV and SRH services among young people. Additionally, as shown in this study, students could benefit from the direct provision of PEP and EC by their peers, and from the inclusion of a PEP‐in‐pocket model. This would strengthen the community‐driven model and is in line with the recently updated WHO PEP guidelines.

Thirty percent of students who collected PEP vouchers redeemed them in this study. This linkage rate is in line with other rates of 21−26% reported for linkage from community HIV testing programmes to health facilities in South Africa [33, 34] and Zimbabwe [35]. Vouchers may have achieved this through providing physical reminders or cues to go to the clinic [36]. Additionally, presenting a voucher meant that the student would then not have to verbally spell out the purpose of the clinic visit, which students would have found helpful in the context of the moral stigma for sexual activity that is described above. The 70% who deemed themselves at risk of HIV acquisition but did not redeem their vouchers represent a group with concerning unmet HIV prevention need. Implementation of the WHO guidelines for PEP has the potential to bridge this gap, and it will be useful for programmes to adopt WHO guidelines that endorse the use of HIVST for PEP and PrEP [37]. HIVST will make it easier to implement community‐driven PEP models and address barriers related to repeated testing that were reported in this study.

Of note, there were indications of actual or hypothetical risk compensation with the use of PEP and EC. Risk compensation has been reported in other HIV prevention programmes where increases in STI have been reported following the implementation of PrEP programmes [38, 39]. Although the data are conflicting, there is evidence that STI rates were rising before PrEP, and there is modelling evidence to show the gains, at least for HIV, far outweigh the problems caused by risk compensation [39]. Further research is needed to quantify the magnitude and impact of the problem in this population and to explore interventions that address it.

Results of our economic cost analysis show that promoting self‐care among college/university students is affordable at US$10−$14 per participant, falling within the range of reported costs of peer‐led distribution programmes across high HIV prevalence settings in Africa varying between US$4 per participant reached to US$36 per HIVST kit pack distributed [40, 41]. Personnel costs account for more than two‐thirds of costs reflecting the intensive nature of supervision and support provided to peer distributors and potentially a source of cost reductions as programmes mature and less support is required or with higher distribution numbers leading to economies of scale [35, 42].

The strengths of our mixed‐methods study include the fact that together with students and MOH we developed a context‐relevant intervention, including a costing study. We used robust methods in intervention development and evaluation. We provide timely evidence to inform the operationalization of recent WHO PEP guidelines on community‐based provision and task sharing. Limitations relate to the small size of the pilot, limiting generalizability and requiring caution in the interpretation of results of the logistic regression. We did not collect data on adherence or completion rates for PEP. The pilot was short in duration, which limits understanding of how evaluated outcomes change with time. Limitations also relate to the accuracy of self‐reports on time since condomless sex, which would be expected for a study of this nature.

## 5 CONCLUSIONS

In conclusion, this mixed‐methods evaluation of a co‐developed peer‐led intervention for HIVST, PEP and EC found that the intervention was acceptable, feasible, appropriate and implemented as intended. The costs of intervention development and implementation were in line with those of similar interventions, with potential cost reduction for large programmes benefiting from economies of scale. Although young people are at an ongoing risk of HIV, STIs and unintended pregnancy, access to relevant HIV and SRH services is limited. Factors such as stigma and unfriendly HWs limit the uptake of HIV and SRH services. The study highlights an urgent need for addressing the challenges we uncovered to drive the attainment of health targets now and in the future. A larger, comparative evaluation of the intervention that is refined as suggested here is needed to evaluate its impact.

## COMPETING INTERESTS

The authors declared no competing interests.

## AUTHORS’ CONTRIBUTIONS

OK: Writing original draft, formal analysis, visualization. LM: Writing original draft, project administration, formal analysis. CM: Writing original draft, methodology, formal analysis. MKT: Project administration, writing—review and editing. NR: Investigation, writing—review and editing. EM: Data curation. JK: Conceptualization. FT‐P: Conceptualization, methodology, writing—review and editing. FMC: Conceptualization, methodology, writing—review and editing, supervision. ELS: Conceptualization, writing an original draft, methodology, funding acquisition, supervision.

## FUNDING

This study was funded by UNAIDS.

## Supporting information




**Table S1**: Characteristics of students taking up commodities by institution

## Data Availability

Data are available upon request. De‐identified data are available from the corresponding author on request.

## References

[1] UNAIDS . Community‐led AIDS responses: final report based on the recommendations of the multistakeholder task team. 2022.

[2] UNAIDS . Global AIDS strategy 2021–2026. End inequalities. End AIDS. 2021.

[3] Joint United Nations Programme on HIV/AIDS (UNAIDS) . IN DANGER: UNAIDS Global AIDS Update 2022. Geneva: UNAIDS; 2022.

[4] Giguère K , Eaton JW , Marsh K , Johnson LF , Johnson CC , Ehui E , et al. Trends in knowledge of HIV status and efficiency of HIV testing services in sub‐Saharan Africa, 2000–20: a modelling study using survey and HIV testing programme data. Lancet HIV. 2021;8(5):e284–e93. 33667411 10.1016/S2352-3018(20)30315-5 PMC8097636

[5] UNAIDS . 2020 Global AIDS Update. 2020.

[6] UNAIDS . 2021 Global AIDS Update: confronting inequalities ‐ lessons for pandemic responses from 40 years of AIDS. 2021.

[7] UNAIDS . The path that ends AIDS: 2023 UNAIDS Global AIDS update. Geneva: UNAIDS; 2023.

[8] PHIA Project . Zimbabwe Population‐based HIV Impact Assessment [ZIMPHIA 2020]. 2021. https://phia.icap.columbia.edu/zimbabwe2020‐final‐report/.

[9] Zimbabwe National Statistics Agency (ZIMSTAT) . Demographic and Health Survey 2023–24: Key Indicators. Rockville, MD: Zimbabwe National Statistics Agency (ZIMSTAT).

[10] McCurdy RJ , Jiang X , Schnatz PF . Long‐acting reversible contraception in adolescents in sub‐Saharan Africa: evidence from demographic and health surveys. Eur J Contracept Reprod Health Care. 2018;23(5):357–64. 30465692 10.1080/13625187.2018.1519535

[11] Nathan SF , Berglas NF , Kaller S , Mays A , Biggs MA . Reasons for having unprotected sex among adolescents and young adults accessing reproductive health services. Women's Health Issues. 2023;33(3):222–7. 36543704 10.1016/j.whi.2022.11.006

[12] UNFPA . Zimbabwe commemorates World Contraception Day with milestone achievement. 2023. (accessed 12 February 2025). https://zimbabwe.unfpa.org/en/news/zimbabwe-commemorates-world-contraception-day-milestone-achievement#:~:text=As%20a%20result%20of%20Government's,per%20cent%20as%20of%202021.

[13] World Health Organisation . Guidelines for HIV post‐exposure prophylaxis. Geneva: WHO; 2023. 39259822

[14] Tumushime MK , Takaruza A , Ruhode N , Chidhanguro K , Masiyambiri F , Mancitshana L , Sibanda EL , et al. Piloting interventions to increase uptake of HIV self‐testing and linkage to post‐services among tertiary education students in Zimbabwe. AIDS 2024, poster presentation at the 25th International AIDS Conference. Munich, Germany; 2024.

[15] World Health Organisation . WHO guideline on self‐care interventions for health and well‐being, 2022 revision. 2022. (accessed 28 August 2024 2024). https://www.who.int/publications/i/item/9789240052192

[16] World Health Organisation . WHO consolidated guideline on self‐care interventions for health. 2021.

[17] World Health Organisation . WHO consolidated guideline on self‐care interventions for health: sexual and reproductive health and rights. 2019. 31334932

[18] World Health Organisation . Implementation of self‐care interventions for health and well‐being: guidance for health systems. 2024.

[19] Narasimhan M , Allotey P , Hardon A . Self care interventions to advance health and wellbeing: a conceptual framework to inform normative guidance. BMJ. 2019;365:l688. 30936087 10.1136/bmj.l688 PMC6441866

[20] Proctor E , Silmere H , Raghavan R , Hovmand P , Aarons G , Bunger A , et al. Outcomes for implementation research: conceptual distinctions, measurement challenges, and research agenda. Adm Policy Ment Health. 2011;38(2):65–76. 20957426 10.1007/s10488-010-0319-7 PMC3068522

[21] StataCorp . Stata Statistical Software: Release 18. College Station, TX: StataCorp LLC; 2023.

[22] Rahimi S , Khatooni M . Saturation in qualitative research: an evolutionary concept analysis. Int J Nurs Stud Adv. 2024;6:100174. 38746797 10.1016/j.ijnsa.2024.100174 PMC11080421

[23] Green J , Browne J . Principles of social research. London: Oxford University Press; 2005.

[24] QSR International . NVIVO 11. 2017 (accessed November 2020). https://lumivero.com/products/nvivo/

[25] McGee K , Sprague L , Indravudh P , Nassif E , Terris‐Prestholt F . Clarifying community engagement: a framework for qualifying the community‐led HIV response. UNAIDS Working Paper. 2024.

[26] Li W , Chu J , Zhu Z , Li X , Ge Y , He Y , et al. Epidemiological characteristics of HIV infection among college students in Nanjing, China: a cross‐sectional survey. BMJ Open. 2020;10(5):e035889. 10.1136/bmjopen-2019-035889 PMC7228536 32404394

[27] Milic M , Gazibara T , Dotlic J , Katanic N , Filimonovic J , Mitic K , et al. Risk perception about HIV among university students in one of the last hotspots for HIV transmission in Europe. J Epidemiol Glob Health. 2023;13(4):794–806. 37728721 10.1007/s44197-023-00151-y PMC10686924

[28] Segawa I , Bakeera‐Kitaka S , Ssebambulidde K , Muwonge TR , Oriokot L , Ojiambo KO , et al. Factors associated with HIV self‐testing among female university students in Uganda: a cross‐sectional study. AIDS Res Ther. 2022;19(1):59. 36457098 10.1186/s12981-022-00484-x PMC9713199

[29] Sulemana A , Abu M , Yidana Z , Apraku E , Iddrisu M , Badasu D . Young people's experiences in accessing sexual and reproductive health services in sub‐Saharan Africa from 1994 to 2019 ‐ a content analysis. Int J Sex Reprod Health Care. 2020;3:017–26.

[30] UNAIDS . Putting young key populations first: HIV and young people from key populations in the Asia and Pacific region. Geneva; 2022.

[31] Chipako I , Singhal S , Hollingsworth B . Factors associated with long‐acting reversible contraceptives usage among sexually active adolescent girls and young women in Zimbabwe. PLOS Glob Public Health. 2024;4(8):e0003551. 39163319 10.1371/journal.pgph.0003551 PMC11335097

[32] World Health Organisation . Alarming decline in adolescent condom use, increased risk of sexually transmitted infections and unintended pregnancies, reveals new WHO report. 2024. (accessed 8 September 2024). https://www.who.int/europe/news/item/29‐08‐2024‐alarming‐decline‐in‐adolescent‐condom‐use–increased‐risk‐of‐sexually‐transmitted‐infections‐and‐unintended‐pregnancies–reveals‐new‐who‐report#:~:text=Decline%20in%20condom%20use%3A%20the,girls%20between%202014%20and%202022

[33] Sharma M , Ying R , Tarr G , Barnabas R . Systematic review and meta‐analysis of community and facility‐based HIV testing to address linkage to care gaps in sub‐Saharan Africa. Nature. 2015;528(7580):S77–85. 26633769 10.1038/nature16044 PMC4778960

[34] Maughan‐Brown B , Beckett S , Kharsany ABM , Cawood C , Khanyile D , Lewis L , et al. Poor rates of linkage to HIV care and uptake of treatment after home‐based HIV testing among newly diagnosed 15‐to‐49 year‐old men and women in a high HIV prevalence setting in South Africa. AIDS Care. 2021;33(1):70–9. 32036678 10.1080/09540121.2020.1719025 PMC7477751

[35] Sibanda EL , Mangenah C , Neuman M , Tumushime M , Watadzaushe C , Mutseta MN , et al. Comparison of community‐led distribution of HIV self‐tests kits with distribution by paid distributors: a cluster randomised trial in rural Zimbabwean communities. BMJ Glob Health. 2021;6(Suppl 4):e005000. 10.1136/bmjgh-2021-005000 PMC8287604 34275872

[36] McLean S , Gee M , Booth A , Salway S , Nancarrow S , Cobb M , et al. Targeting the Use of Reminders and Notifications for Uptake by Populations (TURNUP): a systematic review and evidence synthesis. Southampton: NIHR Journals Library; 2014. 25642537

[37] PrEP Watch . HIV self‐testing and PrEP: opportunities for scale‐up. 2024. (accessed 8 September 2024). https://www.prepwatch.org/resources/hiv‐self‐testing‐and‐prep‐opportunities‐for‐scale‐up/

[38] Milam J , Jain S , Dubé MP , Daar ES , Sun X , Corado K , et al. Sexual risk compensation in a pre‐exposure prophylaxis demonstration study among individuals at risk of HIV. J Acquir Immune Defic Syndr. 2019;80(1):e9–e13. 30334877 10.1097/QAI.0000000000001885 PMC6289757

[39] von Schreeb S , Pedersen SK , Christensen H , Jørgsensen KM , Harritshøj LH , Hertz FB , et al. Questioning risk compensation: pre‐exposure prophylaxis (PrEP) and sexually transmitted infections among men who have sex with men, capital region of Denmark, 2019 to 2022. Eurosurveillance. 2024;29(13):2300451. 38551099 10.2807/1560-7917.ES.2024.29.13.2300451 PMC10979528

[40] Shahmanesh M , Mthiyane TN , Herbsst C , Neuman M , Adeagbo O , Mee P , et al. Effect of peer‐distributed HIV self‐test kits on demand for biomedical HIV prevention in rural KwaZulu‐Natal, South Africa: a three‐armed cluster‐randomised trial comparing social networks versus direct delivery. BMJ Glob Health. 2021;6(Suppl 4):e004574. 10.1136/bmjgh-2020-004574 PMC8317107 34315730

[41] Torres‐Rueda S , Ferrari G , Orangi S , Hitimana R , Daviaud E , Tawiah T , et al. What will it cost to prevent violence against women and girls in low‐ and middle‐income countries? Evidence from Ghana, Kenya, Pakistan, Rwanda, South Africa and Zambia. Health Policy Plan. 2020;35(7):855–66. 32556173 10.1093/heapol/czaa024 PMC7487331

[42] Bernet PM , Singh S . Economies of scale in the production of public health services: an analysis of local health districts in Florida. Am J Public Health. 2015;105 Suppl 2(Suppl 2):S260–7. 25689207 10.2105/AJPH.2014.302350 PMC4355699

