# Supplementary material for: Developing and evaluating a community‐driven intervention to promote uptake of HIV and contraception services among students enrolled in colleges and universities in Zimbabwe
Source: J Int AIDS Soc. 2025 Jun 26;28(Suppl 1):e26461. doi: 10.1002/jia2.26461 (PMC12231659; doi:10.1002/jia2.26461)
Supplement: Supplementary file 1 — Table S1: Characteristics of students taking up commodities by institution [file JIA2-28-e26461-s001.docx]

**Supplementary Table: Characteristics of students taking up commodities by institution**

| **Institution** | **Characteristics** | **Polytechnic College** | **University** | **Vocational College** | **All colleges/ universities** |
| --- | --- | --- | --- | --- | --- |
|  |  | **n (n/N%)** | **n (n/N%)** | **n (n/N%)** | **n (n/N%)** |
| **Male condom**  **(N=473)** | **Age range (years)** |  |  |  |  |
|  | 16-19 | 18 (10.4) | 10 (5.7) | 34 (26.8) | 62(13.1) |
|  | 20-24 | 100 (57.8) | 145 (83.9) | 82 (64.6) | 327 (69.1) |
|  | 25-29 | 50 (28.9) | 17 (9.8) | 10 (7.8) | 77 (16.3) |
|  | 30+ | 5 (2.9) | 1 (0.6) | 1 (0.8) | 7 (1.5) |
|  | Missing | 0 (0.0) | 0 (0.0) | 0 (0.0) | 0 (0.0) |
|  | **Total** | **173 (100.0)** | **173 (100.0)** | **127 (100.0)** | **473 (100.0)** |
|  | **Sex** |  |  |  |  |
|  | Male | 155 (89.6) | 157 (90.8) | 106 (83.5) | 418 (88.4) |
|  | Female | 18 (10.4) | 16 (9.2) | 13 (10.2) | 47 (9.9) |
|  | Missing | 0 (0.0) | 0 (0.0) | 8 (6.3) | 8 (1.7) |
|  | **Total** | **173 (100.0)** | **173 (100.0)** | **127 (100.0)** | **473 (100.0)** |
|  | **Level in college** |  |  |  |  |
|  | First | 31 (17.9) | 47 (27.2) | 76 (59.8) | 154 (32.6) |
|  | Second | 65 (37.6) | 64 (37.0) | 24 (18.9) | 153 (32.3) |
|  | Third | 72 (41.6) | 36 (20.8) | 18 (14.2) | 126 (26.7) |
|  | Fourth and over | 4 (2.3) | 26 (15.0) | 0 (0.0) | 30 (6.3) |
|  | Missing | 1 (0.6) | 0 (0.0) | 9 (7.1) | 10 (2.1) |
|  | **Total** | **173 (100.0)** | **173 (100.0)** | **127 (100.0)** | **473 (100.0)** |
| **Female condom**  **(N=107)** | **Age range (years)** |  |  |  |  |
|  | 16-19 | 6 (10.9) | 2 (4.9) | 8 (72.7) | 16 (15.0) |
|  | 20-24 | 39 (70.9) | 36 (87.8) | 3 (27.3) | 78 (72.9) |
|  | 25-29 | 10 (18.2) | 3 (7.3) | 0 (0.0) | 13 (12.1) |
|  | 30+ | 0 (0.0) | 0 (0.0) | 0 (0.0) | 0 (0.0) |
|  | Missing | 0 (0.0) | 0 (0.0) | 0 (0.0) | 0 (0.0) |
|  | **Total** | **55 (100.0)** | **41 (100.0)** | **11 (100.0)** | **107 (100.0)** |
|  | **Sex** |  |  |  |  |
|  | Male | 8 (14.5) | 12 (29.3) | 5 (45.5) | 25 (23.4) |
|  | Female | 47 (85.5) | 29 (70.7) | 6 (54.5) | 82 (76.6) |
|  | Missing | 0 (0.0) | 0 (0.0) | 0 (0.0) | 0 (0.0) |
|  | **Total** | **55 (100.0)** | **41 (100.0)** | **11 (100.0)** | **107 (100.0)** |
|  | **Level in college** |  |  |  |  |
|  | First | 14 (25.4) | 13 (31.7) | 7 (63.6) | 34 (31.8) |
|  | Second | 26 (47.3) | 14 (34.1) | 4 (36.4) | 44 (41.1) |
|  | Third | 14 (25.5) | 8 (19.6) | 0 (0.0) | 22 (20.6) |
|  | Fourth and over | 1 (1.8) | 6 (14.6) | 0 (0.0) | 7 (6.5) |
|  | Missing | 0 (0.0) | 0 (0.0) | 0(0.0) | 0 (0.0) |
|  | **Total** | **55 (100.0)** | **41 (100.0)** | **11 (100.0)** | **107 (100.0)** |
| **HIVST**  **(N=616)** | **Age range (years)** |  |  |  |  |
|  | 16-19 | 38 (11.2) | 22 (13.6) | 31 (26.7) | 91 (14.8) |
|  | 20-24 | 190 (56.2) | 121 (74.6) | 71 (61.3) | 382 (62.0) |
|  | 25-29 | 102 (30.2) | 16 (9.9) | 12 (10.3) | 130 (21.1) |
|  | 30+ | 8 (2.4) | 3 (1.9) | 2 (1.7) | 13 (2.1) |
|  | Missing | 0 (0.0) | 0 (0.0) | 0 (0.0) | 0 (0.0) |
|  | **Total** | **338 (100.0)** | **162 (100.0)** | **116 (100.0)** | **616 (100.0)** |
|  | **Sex** |  |  |  |  |
|  | Male | 156 (46.2) | 70 (43.2) | 81 (69.8) | 307 (49.8) |
|  | Female | 182 (53.8) | 92 (56.8) | 35 (30.2) | 309 (50.2) |
|  | Missing | 0 (0.0) | 0 (0.0) | 0 (0.0) | 0 (0.0) |
|  | **Total** | **338 (100.0)** | **162 (100.0)** | **116 (100.0)** | **616 (100.0)** |
|  | **Level in college** |  |  |  |  |
|  | First | -* | -* | -* | -* |
|  | Second | -* | -* | -* | -* |
|  | Third | -* | -* | -* | -* |
|  | Fourth and over | -* | -* | -* | -* |
|  | Missing | -* | -* | -* | -* |
|  | **Total** | **-*** | -* | **-*** | -* |
| **PEP** | **Age range (years)** |  |  |  |  |
| **Voucher** | 16-19 | 0 (0.0) | 2 (4.0) | 1 (20.0) | 3 (3.0) |
| **(N=100)** | 20-24 | 23 (51.1) | 38 (76.0) | 2 (40.0) | 63 (63.0) |
|  | 25-29 | 16 (35.6) | 9 (18.0) | 1 (20.0) | 26 (26.0) |
|  | 30+ | 0 (0.0) | 0 (0.0) | 0 (0.0) | 0 (0.0) |
|  | Missing | 6 (13.3) | 1 (2.0) | 1 (20.0) | 8 (8.0) |
|  | **Total** | **45 (100.0)** | **50 (100.0)** | **5 (100.0)** | **100 (100.0)** |
|  | **Sex** |  |  |  |  |
|  | Male | 13 (28.9) | 28 (56.0) | 0 (0.0) | 41 (41.0) |
|  | Female | 26 (57.8) | 21 (42.0) | 4 (80.0) | 51 (51.0) |
|  | Missing | 6 (13.3) | 1 (2.0) | 1 (20.0) | 8 (8.0) |
|  | **Total** | **45 (100.0)** | **50 (100.0)** | **5 (100.0)** | **100 (100.0)** |
|  | **Level in college** |  |  |  |  |
|  | First | 12 (26.7) | 5 (10.0) | 2 (40.0) | 19 (19.0) |
|  | Second | 10 (22.2) | 23 (46.0) | 0 (0.0) | 33 (33.0) |
|  | Third | 15 (33.3) | 6 (12.0) | 2 (40.0) | 23 (23.0) |
|  | Fourth and over | 0 (0.0) | 12 (24.0) | 0 (0.0) | 12 (12.0) |
|  | Missing | 8 (17.8) | 4 (8.0) | 1 (20.0) | 13 (13.0) |
|  | **Total** | **45 (100.0)** | **50 (100.0)** | **5 (100.0)** | **100 (100.0)** |
| **EC Voucher** | **Age range (years)** |  |  |  |  |
| **(N=257)** | 16-19 | 5 (6.2) | 4 (8.3) | 48 (37.5) | 57 (22.2) |
|  | 20-24 | 32 (39.5) | 35 (72.9) | 68 (53.1) | 135 (52.5) |
|  | 25-29 | 13 (16.1) | 9 (18.8) | 11 (8.6) | 33 (12.8) |
|  | 30+ | 0 (0.0) | 0 (0.0) | 1 (0.8) | 1 (0.4) |
|  | Missing | 31 (38.2) | 0 (0.0) | 0 (0.0) | 31 (12.1) |
|  | **Total** | **81 (100.0)** | **48 (100.0)** | **128 (100.0)** | **257 (100.0)** |
|  | **Sex** |  |  |  |  |
|  | Male | 1 (1.2) | 16 (33.3) | 101 (78.9) | 118 (45.9) |
|  | Female | 49 (60.5) | 32 (66.7) | 27 (21.1) | 108 (42.0) |
|  | Missing | 31 (38.3) | 0 (0.0) | 0 (0.0) | 31 (12.1) |
|  | **Total** | **81 (100.0)** | **48 (100.0)** | **128 (100.0)** | **257 (100.0)** |
|  | **Level in college** |  |  |  |  |
|  | First | 12 (14.8) | 15 (31.3) | 82 (64.1) | 109 (42.4) |
|  | Second | 24 (29.7) | 9 (18.8) | 31 (24.2) | 64 (24.9) |
|  | Third | 13 (16.0) | 6 (12.5) | 14 (10.9) | 33 (12.8) |
|  | Fourth and over | 0 (0.0) | 16 (33.4) | 1 (0.8) | 17 (6.6) |
|  | Missing | 32 (39.5) | 2 (4.2) | 0 (0.0) | 34 (13.3) |
|  | **Total** | **81 (100.0)** | **48 (100.0)** | **128 (100.0)** | **257 (100.0)** |

*Foot note: -* indicates that the tool used for documenting HIVST distribution did not collect data on level in college*
